# PW02-033 - Cytokine profile in CSF in CAPS patients

**DOI:** 10.1186/1546-0096-11-S1-A174

**Published:** 2013-11-08

**Authors:** E Schuh, P Lohse, M Frankenberger, I Meinl, T Kuempfel

**Affiliations:** 1 Neurology, Institut of Clinical Neuroimmunology, Munich, Germany; 2 Laboraroriumsmedizin, Singen; 3 Helmholtz Zentrum, Munich, Germany

## Introduction

CAPS is a rare autoinflammatory syndrome caused by autosomal dominant mutations in the NLRP3/CIAS 1 gene on chromosome 1q44 encoding for the cryopyrin protein, an important component of the inflammasome, leading to excessive production of interleukin-1beta (IL-1ß). CAPS encompasses three different entities of variable clinical severity: familial cold auto-inflammatory syndrome (FCAS), Muckle-Wells syndrome (MWS) and chronic infantile neurological cutaneous articular syndrome (CINCA)/ neonatal – onset multisystem inflammatory disease (NOMID). They are all characterised by recurrent episodes of systemic inflammation involving particularly skin, joints, central nervous system and eyes.

## Objectives

To analyse and quantify various cytokines in sera and cerebrospinal fluid (CSF) in five patients with central nervous system (CNS) manifestations of cryopyrin-associated periodic syndromes (CAPS) carrying the Q703K mutation in heterozygosity.

## Methods

Five Caucasian patients (mean age 37 ±10 years; one male) with CNS manifestations including optic nerve inflammation, recurrent cranial nerve palsy, migraine, fatigue, and recurrent meningitis were identified as heterozygous carriers of the cryopyrin Q703K substitution. CSF investigations were performed for diagnostic purposes in all patients and showed pleocytosis in 3 patients. In addition, concentrations of the proinflammatory cytokines interleukin beta (IL-1), interleukin-6 (IL-6), interleukin 17 (IL-17), tumor necrosis factor alpha (TNF-alpha) and FGF (Fibroblast growth factor) were determined in the CSF and sera using a multiplex assay.

## Results

IL-6 concentrations in the CSF were clearly elevated in two patients during acute attacks of CAPS-associated CNS manifestations. The other three patients were investigated during remission and showed no IL-6 elevations in the CSF. The other serum cytokine levels were increased in one patient.

## Conclusion

Our results show a correlation between CSF IL-6 concentrations and CAPS-associated disease activity in the CNS. IL-6 levels in the CSF therefore may serve as a marker of disease activity in CAPS patients with CNS manifestations.

## Competing interests

E. Schuh: None declared, P. Lohse: None declared, M. Frankenberger: None declared, I. Meinl: None declared, T. Kuempfel Grant / Research Support from: Novartis

